# Coming of Age—Sexual Reproduction in *Candida* Species

**DOI:** 10.1371/journal.ppat.1001155

**Published:** 2010-12-23

**Authors:** Richard J. Bennett

**Affiliations:** Department of Molecular Microbiology and Immunology, Brown University, Providence, Rhode Island, United States of America; University of California San Francisco, United States of America

## What Are *Candida* Species and Why Are They Important?

Historically, *Candida* species represented a catch-all taxonomic grouping for yeasts that exhibited hyphal or pseudohyphal branching and did not form sexual spores. However, with the advent of molecular typing of DNA sequences, it is now apparent that *Candida* represent a diverse range of species within the Hemiascomycetes, which also includes the model yeast *Saccharomyces cerevisiae*. Several *Candida* species are prominent human pathogens, including *Candida glabrata, Candida parapsilosis*, and *Candida albicans*, the latter being the most common human pathogenic fungus. While *C. albicans* is a natural commensal, typically found amongst the microbiota inhabiting the gastrointestinal tract, it is also the cause of debilitating mucosal infections as well as life-threatening systemic infections. Taken together, *Candida* species are the fourth most common cause of nosocomial bloodstream infections, where they account for 8%–10% of such infections [1].

**Figure 1:**
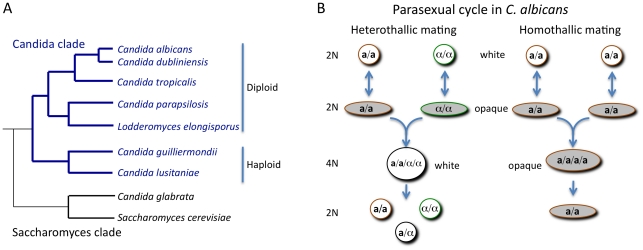
Phylogeny of the *Candida* clade and the parasexual mating cycle of *Candida albicans*. (A) Phylogenetic organization of the *Candida* clade of species and its relationship to the *Saccharomyces* clade. Most *Candida* species belong to the *Candida* clade, including the most frequently isolated species, *C. albicans*. A notable exception is the pathogen *C. glabrata*, which is more closely related to *S. cerevisiae* than to other *Candida* species. (B) Heterothallic and homothallic mating cycles of *C. albicans*. In both mating cycles cells must switch from the white state to the opaque state to become mating competent. Heterothallic mating involves fusion of diploid **a** and α cells to form a white tetraploid **a**/α cell. Under some conditions, homothallic mating can occur with between two opaque **a** cells (or two α cells) to form an opaque tetraploid cell.

The most clinically important *Candida* species, with the exception of *C. glabrata* and *Candida krusei*, cluster together in a single clade [2]. The species within this clade share an altered genetic code in which CUG codons encode serine rather than the universal leucine [3]. The *Candida* clade species can be further subdivided into two separate sub-clades; one contains haploid species that are relatively rare pathogens (e.g., *Candida lusitaniae*), while the other contains diploid species such as *C. albicans* and *C. parapsilosis* that are frequent pathogens (Figure 1A).

## How Was Mating Discovered in *C. albicans*?

Sexual reproduction in fungi is often regulated by genes encoded at a mating-type locus, as exemplified by the *MAT* locus of *S. cerevisiae*. *MAT* **a**-containing cells mate with *MAT*α-containing cells in a program choreographed by *MAT*-encoded transcription factors. The discovery of a mating-type–like (*MTL*) locus in *C. albicans* [4] provided the impetus for re-examining the potential for sexual reproduction in this species. Subsequent experiments in both the Johnson and Magee groups revealed that diploid *MTL* **a** and *MTL*α strains could indeed be made to mate and form stable tetraploids, either on laboratory media or during bloodstream infection of a mammalian host [5], [6]. Despite uncovering mating in the “asexual” *C. albicans*, the frequency of cell–cell conjugation observed was extremely low. This conundrum was solved when it was discovered that *C. albicans* mating is regulated by a unique mechanism of phenotypic switching. First described by Soll and colleagues, some isolates of *C. albicans* had been noted for their ability to undergo switching between “white” and “opaque” states [7]. White cells are round and give rise to shiny, domed-shaped colonies while opaque cells are more elongated and produce darker, flatter colonies. Miller and Johnson noted that only **a** or α strains (and not **a**/α strains) could undergo switching to the opaque form. Furthermore, opaque cells were demonstrated to mate a million times more efficiently than white cells (Figure 1B) [8].

Completion of fungal sexual cycles is usually accomplished via meiosis, in which one round of DNA replication precedes two rounds of DNA division. In the case of *C. albicans*, many potential meiotic genes were found in the sequenced genome but no experimental evidence for a conventional meiosis exists [9]. Instead, a genetic screen showed that a parasexual mechanism of chromosome loss could complete the mating cycle. Growth of *C. albicans* tetraploids on selective media caused random, but concerted, chromosome loss resulting in diploid (and aneuploid) products [10]. A subset of parasexual cells was analyzed and shown to have undergone inter-chromosomal recombination, indicating that the parasexual cycle generates recombinant forms of the species with potentially novel properties [11].

## What Is the Mechanism of White–Opaque Phenotypic Switching?

Recent studies have begun to shed light on the molecular mechanism underlying this bistable switch that regulates pleiotropic aspects of *C. albicans* biology. In addition to mating, the white–opaque switch influences the expression of a number of metabolic genes, determines how *C. albicans* cells interact with host immune cells, and also modulates the virulence of strains during host infection [12]–[14]. It is now established that the master regulator of the opaque state is the transcription factor Wor1p. High levels of Wor1p cause switching to opaque, and Wor1p binding to its own promoter drives a positive feedback loop that stabilizes the opaque form [15]–[17]. Wor1p appears to be representative of a novel superfamily of DNA-binding proteins that is conserved across all fungi [18]. Three additional transcription factors (Efg1p, Czf1p, and Wor2p) complete a network of complementary transcriptional feedback loops with Wor1p that generates the two alternative phenotypic states [19].

In addition to new mechanistic insights into switching, several studies have illustrated the sensitivity of the white–opaque switch to multiple external stimuli. A growing list of conditions that promote switching to opaque includes anaerobic culture, high levels of CO_2_, N-acetyl glucosamine (also a product of commensal bacteria), oxidative stress, and slower growth of the cell [20]–[23]. Significantly, some environmental conditions can stabilize the opaque form at 37°C, a temperature that normally results in conversion en masse of opaque cells to white cells [21]. The fact that opaque cells can persist in certain host niches is supported by observations of white-to-opaque switching in the gastrointestinal tract by some strains of *C. albicans* [23].

**Figure 2:**
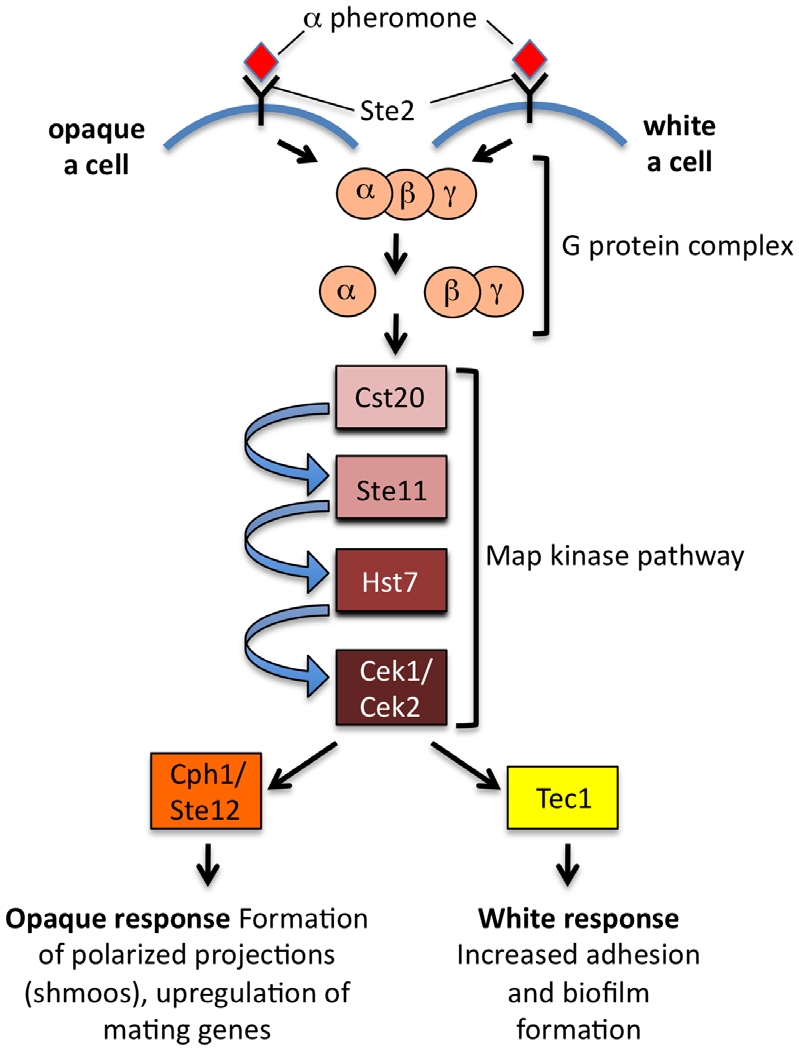
Differential response of *C. albicans* white and opaque cells to pheromone. Both cell types respond to pheromone using the conserved pheromone MAP kinase cascade, but opaque cells use the Ste12/Cph1 transcription factor to turn on genes necessary for cell–cell conjugation, whereas white cells use the Tec1 transcription factor to turn on genes involved in adherence and biofilm formation. Coordination of these pathways may promote mating in vivo. Adapted from Sahni et al. [26].

The central question remains as to why the white–opaque switch evolved to regulate mating in *C. albicans*. It is possible that precise regulation of mating is required to limit it to specific niches in the host. Opaque cells are also targeted less efficiently by host immune cells than white cells, perhaps shielding mating events from destruction [24]. In addition, it is now apparent that white cells, although incapable of mating, could play a key role in sexual reproduction. While opaque cells respond to secreted pheromones by forming mating projections and undergoing cell–cell fusion, white cells exhibit a very different response in which they display increased adhesion to each other and to synthetic surfaces [25]. The ability of white cells to form such pheromone-induced biofilms could promote mating of opaque cells by allowing stable diffusion of pheromone gradients across relatively large distances. Strikingly, the differential response of white and opaque cells involves distinct transcription factors activated by the pheromone mitogen-activated protein (MAP) kinase cascade; this conserved signaling pathway activates the Ste12/Cph1 transcription factor in opaque cells, yet acts via the Tec1 transcription factor in white cells (Figure 2) [26]. It remains to be seen how these two different transcription factors are targeted by the same MAP kinase cascade, although multiple mechanisms help prevent cross-talk between related MAP kinase signaling pathways in *S. cerevisiae* [27].

## How Does *C. albicans* Undergo Homothallic Mating?

Heterothallic mating between opaque **a** and α cells is now well established in *C. albicans*, but recent studies demonstrate that homothallic mating, or self-fertilization, can also occur. Self-mating between **a** cells was first detected upon deletion of *BAR1*, a gene encoding a protease activity that degrades α pheromone [28]. In conventional **a**-α mating in yeast, **a** cells secrete **a** pheromone while α cells secrete α pheromone to attract partners of the opposite sex. However, it is now apparent that *C. albicans* **a** cells secrete both **a** and α pheromones; α pheromone is normally degraded by Bar1p, but in the absence of this protease α pheromone accumulates, leading to autocrine signaling and induction of self-mating in unisexual populations of (opaque) **a** cells. The products of same-sex mating are **a**-**a** tetraploid cells that can still undergo the parasexual program of chromosome loss to return to the diploid state [28]. The discovery of same-sex mating in *C. albicans* shows interesting parallels with that previously made in a distantly related fungal pathogen, *Cryptococcus neoformans*. In *C. neoformans*, efficient mating between **a** and α cells can take place, but the predominant mode of mating in nature appears to be unisexual, with α cells self-mating to form α-α cells that can still undergo meiosis and sporulation [29], [30].

Why should such a mechanism of self-fertilization evolve in *C. albicans* and *C. neoformans,* two such diverse species? Several possibilities have been considered, including the fact that self-mating could help maintain the sexual/parasexual machinery for future (rare) heterothallic mating events [30]. In addition, same-sex mating events have the potential to increase genetic diversity as well as generate changes in ploidy that may increase fitness [29]. In the case of *C. albicans*, same-sex mating products can also exist in the opaque state (whereas conventional **a**-α products are obligate white cells), and this may provide additional, yet to be discovered, benefits for the species (see Figure 1B) [28].

## What About Sexual Reproduction in Other *Candida* Species?

With the recent release of genome sequences for multiple *Candida* species [2], new insights into mechanisms of fungal sex are emerging. A conserved *MTL* locus is present in the majority of strains from the *Candida* clade, although surprises have been found in species such as *Lodderomyces elongisporus*. This yeast is thought to be homothallic due to the production of asci-like spores, and yet it has lost all of the conserved transcriptional regulators of sexual identity at the *MTL* [2]. If a sexual cycle does take place in *L. elongisporus* it must therefore be under novel regulatory control. In this regard, it is interesting to note that mating in the basidiomycete *C. neoformans* was recently shown to be regulated by transcription factors that are not encoded at the mating locus [31]. In addition, the re-wiring of sexual transcriptional circuits has occurred multiple times during the evolution of the *Candida* clade. An example of this is seen in *C. lusitaniae*, which exhibits a complete sexual cycle culminating in meiosis and sporulation despite lacking the *MTL* transcription factor α2. Furthermore, *C. lusitaniae* has apparently lost several conserved meiosis factors, including the strand exchange protein Dmc1 and the synaptonemal complex proteins Zip1-4 and Hop1 [32]. Conversely, the asexual species *C. tropicalis* and *C. parapsilosis* do not appear to undergo mating or asci formation, yet contain many of the genes required for mating and meiosis [2]. These observations emphasize that sequence analysis alone is not sufficient to determine whether a species is able to complete a sexual (or parasexual) cycle. It is likely that cryptic mating cycles are still to be discovered in some *Candida* species, while in others, the sexual machinery is under different selective pressures and components have been retained for uses other than mating and recombination. What is certain is that defining the roles of mating and mating-related mechanisms in *Candida*, and their potential function in commensal and infectious growth, will provide very fertile ground in the years to come.
